# Coronary Artery Calcification as a Marker for Coronary Artery Stenosis: Comparing Kidney Failure to the General Population

**DOI:** 10.1016/j.xkme.2021.01.010

**Published:** 2021-04-20

**Authors:** Thijs T. Jansz, Meike H.Y. Go, Nolan S. Hartkamp, J. Lauran Stöger, Csilla Celeng, Tim Leiner, Pim A. de Jong, Frank J.L. Visseren, Marianne C. Verhaar, Brigit C. van Jaarsveld

**Affiliations:** 1 Departments of Nephrology and Hypertension, University Medical Center Utrecht, Utrecht University, Utrecht; 2 Department of Radiology, University Medical Center Utrecht, Utrecht University, Utrecht; 3 On behalf of the UCC-SMART study group, Department of Vascular Medicine, University Medical Center Utrecht, Utrecht University, Utrecht; 4 Department of Radiology, Amsterdam UMC, Amsterdam; 5 Department of Radiology, Leiden University Medical Center, Leiden; 6 Department of Nephrology, Amsterdam UMC, Amsterdam Cardiovascular Sciences, Amsterdam; 7 Dianet Dialysis Centers, Utrecht, the Netherlands

**Keywords:** Coronary calcification, coronary stenosis, ischemic cardiac disease, end-stage kidney disease, renal failure, hemodialysis, peritoneal dialysis

## Abstract

**Rationale & Objective:**

The presence of calcified plaques in the coronary arteries is associated with cardiovascular mortality and is a hallmark of chronic kidney failure, but it is unclear whether this is associated with the same degree of coronary artery stenosis as in patients without kidney disease. We compared the relationship of coronary artery calcification (CAC) and stenosis between dialysis patients and patients without chronic kidney disease (CKD).

**Study Design:**

Observational cohort study.

**Setting & Participants:**

127 dialysis patients and 447 patients without CKD with cardiovascular risk factors underwent cardiac computed tomography (CT), consisting of non–contrast-enhanced CT and CT angiography. CAC score and degree of coronary artery stenosis were assessed by independent readers.

**Predictor:**

Dialysis treatment.

**Outcome:**

Association between calcification and stenosis.

**Analytical Approach:**

Logistic regression to determine the association between CAC score and the presence of stenosis in a matched cohort and, in the full cohort, testing for the interaction of dialysis status with this relationship.

**Results:**

112 patients were matched from each cohort, totaling 224 patients, using propensity scores for dialysis, balancing numerous cardiovascular risk factors. Median CAC score was 210 (IQR, 19-859) in dialysis patients and 58 (IQR, 0-254) in patients without CKD; 35% of dialysis patients and 36% of patients without CKD had coronary artery stenosis ≥ 50%. Per each 100-unit higher CAC score, the matched dialysis cohort had significantly lower ORs for stenosis than the non-CKD cohort, 0.67 (95% CI, 0.52-0.83) for stenosis ≥ 50% and 0.75 (95% CI, 0.62-0.90) for stenosis ≥ 70%.

**Limitations:**

No comparison with the gold standard fractional flow reserve.

**Conclusions:**

Dialysis patients have higher risk for coronary artery stenosis with higher CAC scores, but this risk is comparatively lower than in patients without CKD with similar CAC scores. In dialysis patients, a high CAC score can easily be found without significant stenosis. Our data enable “translation” of degree of calcification to the probability of coronary stenosis in dialysis patients.

## Graphical abstract

**Figure:**
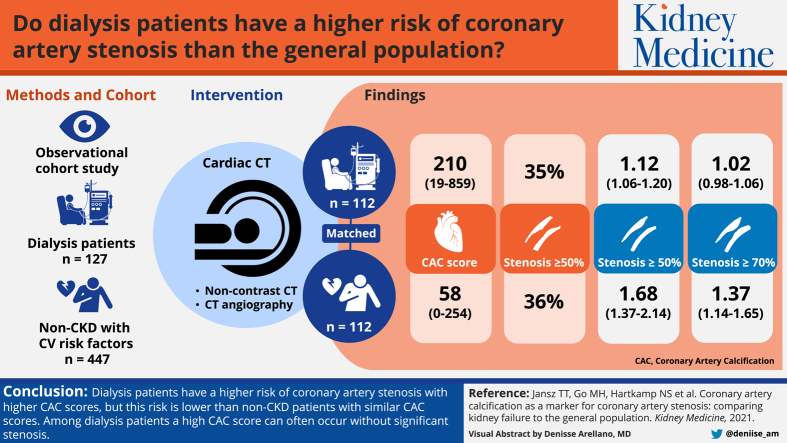

**Plain-Language Summary**

In the general population, an increase in coronary calcification is well associated with coronary artery stenosis. In dialysis patients, vascular calcification is much more pronounced due to metabolic derangements. We quantitatively compared the relation of coronary calcification and stenosis between patients treated with dialysis and patients without chronic kidney disease but with risk factors for atherosclerosis. We found that dialysis patients have a higher probability of coronary artery stenosis with higher calcification levels, but this probability is about 1.5 times lower than in patients without kidney disease having similar calcification levels. In dialysis patients, a high degree of calcification can easily be found without significant stenosis. Our data enable “translation” of degree of calcification to probability of coronary stenosis in dialysis patients.

In the general population, the presence of vascular calcification is one of the main risk factors for mortality because it reflects the overall burden of coronary atherosclerosis and probability for obstruction.^1^, ^2^, ^3^, ^4^, ^5^, ^6^, ^7^ Calcification in patients with end-stage kidney disease (ESKD) is much more extensive than in patients with normal kidney function,^8^^,^^9^ especially when they are treated with dialysis. This is the result of disturbed mineral homeostasis, with a hallmark transformation of vascular smooth muscle cells to osteoblast-like cells, and increased phosphate levels being one of the main drivers of this calcification process.^10^^,^^11^ In ESKD, including both patients requiring dialysis and those with a kidney transplant, vascular calcification strongly predicts all-cause and cardiovascular mortality.^12^, ^13^, ^14^, ^15^

In patients without kidney disease, coronary calcification mostly occurs in the intima of the vessel wall and can lead to arterial stenosis.^16^^,^^17^ This might be different in ESKD, in which vascular calcification occurs mainly in the media of the vessel wall. Medial calcification is more diffuse and nonocclusive, in contrast to intimal calcification.^18^ Consequently, it is unclear whether coronary artery calcification (CAC) is similarly predictive of coronary stenosis in patients with ESKD as it is in the general population.^19^ Unfortunately, a reliable distinction of medial and intimal calcification is not possible in vivo,^18^ leading previous studies on the relative contribution of intimal and medial calcification in patients with ESKD to report conflicting results on this topic. Additionally, these studies involved patients with symptoms of acute coronary syndrome, thus introducing selection bias by increasing the likelihood of stenosis and preferring intimal calcification.^20^^,^^21^ Also, previous studies sometimes used electron beam computed tomography (CT), which has now been replaced by multislice CT; these techniques show a good correlation regarding calcification score, but modern techniques have a higher special resolution and are more suitable for CT angiography.

Our aim was therefore to determine the relationship between CAC and coronary artery stenosis, using coronary CT angiography, in asymptomatic patients with ESKD treated with dialysis and in individuals with normal kidney function and an otherwise similar cardiovascular risk profile.

## Methods

We examined patients with ESKD from the NOCTx Study (NCT00950573) and participants without known chronic kidney disease (CKD) from the Secondary Manifestation of Arterial Disease–Optimizing Risk Assessment for Cardiovascular Events (SMART-ORACLE; NCT01932671) Study. Both studies were approved by the Medical Ethics Committee of the University Medical Center Utrecht (NL18314.041.08) and were conducted according to the Declaration of Helsinki.

### Study Populations

#### Dialysis Cohort

The NOCTx Study is a prospective cohort study that investigated annual progression of CAC in patients treated with different dialysis modalities (nocturnal or conventional hemodialysis, peritoneal dialysis) and kidney transplant recipients. At inclusion, all patients had been receiving dialysis for at least 2 months and underwent coronary CT and angiography. Consequently, all included patients in the present study were treated with and are henceforward referred to as dialysis patients. Dialysis patients aged between 18 and 75 years who were candidates for transplantation were eligible to participate in the study. All patients gave written informed consent. Patients with life expectancy less than 3 months, nonadherence to dialysis regimens, drug abuse, allergy to iodinated contrast, and pregnancy were excluded. Between December 2009 and February 2016, a total of 329 patients were screened for eligibility in 8 Dutch dialysis centers. There were 181 patients who met the inclusion criteria, of whom 127 patients underwent coronary CT angiography at University Medical Center Utrecht, the Netherlands.

#### Non-CKD Cohort

As a reference cohort, we analyzed participants in the ongoing SMART-ORACLE Study, a prospective observational study designed to investigate whether and to what extent risk assessment can be improved with CAC scoring and cardiovascular CT angiography and CAC score in patients at high risk for a cardiovascular event.^22^ Upon inclusion, participants underwent CT and coronary CT angiography. SMART-ORACLE included patients aged between 18 and 75 years with cardiovascular risk factors (including diabetes mellitus, hypertension with blood pressure > 140/90 mm Hg, positive family history, or hypercholesterolemia) or any clinical manifestation of arterial atherosclerosis (coronary artery disease, cerebrovascular disease, peripheral artery disease, or abdominal aortic aneurysm). Patients with terminal malignancy, estimated glomerular filtration rate (eGFR) < 46 mL/min/1.73 m^2^ according to the MDRD (Modification of Diet in Renal Disease) Study equation, allergy to iodinated contrast, and pregnancy were excluded. Between August 2012 and June 2017, a total of 572 patients were included in the study. Coronary CT angiography was performed in patients with CAC scores less than 1,000 Agatston units, which involved 447 patients.

### Cardiac CT

Image acquisition was performed in accordance with the Society of Cardiovascular Computed Tomography guidelines.^23^ If needed, β-blockers were administered to target a heart rate of 60 beats/min. CT was performed using a 256-section CT scanner (Brilliance iCT; Philips Healthcare). Noncontrast prospective electrocardiographically triggered CT was performed to evaluate coronary calcium score on a per–coronary artery basis.

Subsequently, 0.4 mg of nitroglycerine was sublingually administered to all patients. Patients were given 70 to 80 mL of contrast agent (Bayer Healthcare Pharmaceuticals), followed by a 50- to 67-mL mixed contrast/saline solution bolus and 30 to 40 mL of intravenous saline solution (injected at a rate of 6-6.7 mL/s). The contrast/saline solution bolus consisted of a 50%/50% mixture for the dialysis cohort and a 30%/70% mixture for the non-CKD group. Coronary CT angiography was performed by using a prospectively electrocardiographically triggered sequential scan (≤60 beats/min) or retrospectively electrocardiographically gated helical scan (>60 beats/min). Depending on body mass, tube voltage ranged between 80 and 120 kV peak, and tube current, between 200 and 300 mA for prospectively triggered scans and 600 mA for retrospectively gated scans. Images were reconstructed with a section thickness of 0.9 mm and an increment of 0.45 mm using a standard kernel.

### Image Interpretation and Analysis

The coronary artery tree was assessed using the Society of Cardiovascular Computed Tomography segmentation diagram^24^ with dedicated software (Comprehensive Cardiac; Philips Healthcare). Scans were analyzed by 2 readers: 1 with 2 years of experience (T.T.J. or M.H.Y.G.) and 1 with 8 to 12 years of experience (N.S.H., L.L.S., or C.C.). Disagreements were resolved by consensus readings. All readers were blinded to clinical information.

We defined coronary lesions as lesions within or adjacent to the vessel lumen discernable in at least 2 planes from both vessel lumen and adjacent soft tissue. We categorized lesions as noncalcified (content exclusively <130 Hounsfield units [HU]), calcified (content exclusively ≥130 HU), or mixed (characteristics of both noncalcified and calcified lesions). Per segment, we assessed stenosis severity visually as no lesion, minimal or mild (<50%) stenosis, moderate (50%-70%) stenosis, and severe (≥70%) stenosis.

### Other Variables

For dialysis patients, demographics were collected by chart review. Data for predialysis blood pressure and postdialysis weight were averaged from routine measurements during 3 hemodialysis sessions or 2 outpatient visits in case of peritoneal dialysis. Routine laboratory measurements (phosphate, C-reactive protein, and total cholesterol) were performed using standard laboratory techniques.

In the non-CKD group, patients completed a standardized vascular screening protocol,^25^ including a questionnaire on medical history and smoking status and physical examination including office blood pressure. Further data collection and laboratory techniques have been described elsewhere.^25^^,^^26^

### Statistical Analysis

We present data as mean ± standard deviation when normally distributed, median with interquartile range (IQR) when non-normally distributed, and proportion when categorical. Patients from the dialysis and non-CKD cohorts were matched using propensity scores to balance confounding variables.^27^ In the propensity score logit model, we included traditional cardiovascular risk factors, such as age, sex, body mass index, current smoking, presence of diabetes mellitus, and C-reactive protein and total cholesterol levels. We matched nearest neighbors in a 1:1 ratio, without replacement, within a 0.2 caliper. When standardized mean differences were <0.1, we considered covariates balanced. We classified patients according to the presence of stenosis: no significant stenosis, any stenosis ≥ 50%, and any stenosis ≥ 70%. We used logistic regression to determine the relationship of CAC score and presence of any stenosis ≥ 50% or ≥70% in the matched cohort. In a second analysis, we repeated the logistic regression analyses with all patients using a multivariable model adjusted for the same variables as used in the propensity score logit model. In this analysis, we tested for interaction between dialysis and CAC on their effect on stenosis.

Odds ratios were calculated from regression coefficients and reported with 95% CIs and *P*≤0.05 (2 tailed) was considered statistically significant. All calculations were done using R, version 3.4.1 (R Foundation Statistical Computing).

## Results

### Dialysis and Non-CKD Cohorts

In the dialysis cohort (n = 127), mean age was 51.4 years, 86 (68%) were men, and median dialysis duration was 25 (IQR, 12-49) months. At the time of coronary CT angiography, 98 (77%) patients were treated with hemodialysis, and 29 (23%) patients with peritoneal dialysis. Twenty-three (18%) patients had diabetes mellitus, and 15 (12%) had a history of coronary artery disease.

In the non-CKD cohort (n = 447), mean age was 57.2 years, 331 (74%) were men, and eGFR was 91 ± 16 mL/min/1.73 m^2^. Fifty-eight (13%) patients had diabetes mellitus, and 256 (57%) had a history of coronary artery disease (Table 1).

**Table 1:** Characteristics of the 127 Dialysis Patients and 447 Patients Without CKD at Risk for Cardiovascular Disease at Time of Coronary CT Angiography

	Dialysis (n = 127)	Non-CKD (n = 447)	SMD
Demographics and medical history			
Age, y	51.4 ± 13.2	57.2 ± 9.4	0.51
Male sex	86 (68%)	331 (74%)	0.14
Body mass index, kg/m^2^	25.7 ± 4.9	26.7 ± 4.0	0.23
Diabetes mellitus	23 (18%)	58 (13%)	0.14
Cardiovascular disease	27 (21%)	389 (87%)	1.75
Coronary artery disease	15 (12%)	255 (57%)	1.09
Active smoker	17 (13%)	121 (27%)	0.35
History of kidney disease			
Dialysis modality (%)			
Hemodialysis	98 (77%)	—	—
Peritoneal dialysis	29 (23%)	—	—
Dialysis vintage, mo	25 (12–49)	—	—
Cause of end-stage kidney disease			
Cystic kidney disease	17 (13%)	—	—
Interstitial nephritis	2 (2%)	—	—
Glomerulonephritis	34 (27%)	—	—
Vascular disease	30 (24%)	—	—
Diabetic nephropathy	11 (9%)	—	—
Other	20 (16%)	—	—
Unknown	13 (10%)	—	—
Clinical and biochemical parameters			
Systolic blood pressure, mm Hg	141 ± 19	130 ± 15	0.61
Diastolic blood pressure, mm Hg	80 ± 12	79 ± 9	0.14
C-Reactive protein, mg/L	3.0 [2.0-7.0]	1.6 [0.9-3.8]	0.04
Total cholesterol, mmol/L	4.26 ± 1.21	4.61 ± 1.20	0.29
eGFR, mL/min	—	91 ± 16	—
Coronary artery calcification			
Agatston score	152 [0-774]	110 [3-345]	—

*Note:* Results are presented as mean ± standard deviation, median [interquartile range], or number (percent). Covariates were considered balanced when SMDs were <0.1.

Abbreviations and Definitions: CKD, chronic kidney disease; CT, computed tomography; eGFR, estimated glomerular filtration rate, calculated using the Modification of Diet in Renal Disease Study formula; Non-CKD: non–chronic kidney disease at risk for cardiovascular disease; SMD, standardized mean difference.

### Matched Dialysis and Non-CKD Cohorts

We matched 224 patients from both cohorts on propensity scores of dialysis, balancing the cardiovascular risk factors mentioned across the matched dialysis and non-CKD cohorts (Table 2). According to inclusion criteria, a history of cardiovascular disease or coronary artery disease was more prevalent in the matched non-CKD cohort. By nature of the disease, blood pressure and CAC scores were higher in the matched ESKD cohort.

**Table 2:** Characteristics of the 112 Dialysis Patients and 112 Patients Without CKD at Risk for Cardiovascular Disease, Matched on Propensity Scores

	Dialysis (n = 112)	Non-CKD (n = 112)	SMD
Demographics and medical history			
Age, y	54.1 ± 11.1	53.5 ± 9.4	0.06
Male sex	78 (70%)	80 (71%)	0.04
Body mass index, kg/m^2^	26.2 ± 4.9	26.2 ± 4.0	<0.001
Diabetes mellitus	20 (18%)	17 (15%)	0.07
Cardiovascular disease	26 (23%)	100 (89%)	1.79
Coronary artery disease	15 (13%)	58 (52%)	0.90
Active smoker	17 (15%)	20 (18%)	0.07
History of kidney disease			
Treatment with peritoneal dialysis	25 (22%)	—	—
Dialysis vintage, mo	21 [12-48]	—	—
Cause of end-stage kidney disease			
Cystic kidney disease	16 (14%)	—	—
Interstitial nephritis	2 (2%)	—	—
Glomerulonephritis	26 (23%)	—	—
Vascular disease	30 (27%)	—	—
Diabetic nephropathy	9 (8%)	—	—
Other	17 (15%)	—	—
Unknown	12 (11%)	—	—
Clinical and biochemical parameters			
Systolic blood pressure, mm Hg	141 ± 19	128 ± 14	0.77
Diastolic blood pressure, mm Hg	80 ± 12	79 ± 9	0.17
C-Reactive protein, mg/L	3.0 [2.0-7.0]	1.4 [0.9-3.2]	0.09
Total cholesterol, mmol/L	4.35 ± 1.26	4.41 ± 1.13	0.06
eGFR, mL/min	—	93 ± 16	—
Coronary artery calcification			
Agatston score	210 (19–859)	58 (0–254)	—

*Note:* Results are presented as mean ± standard deviation, median [interquartile range], or number (percent). Covariates were considered balanced when SMDs were <0.1.

Abbreviations and Definitions: CKD, chronic kidney disease; eGFR, estimated glomerular filtration rate, calculated using the Modification of Diet in Renal Disease Study formula; Non-CKD: non–chronic kidney disease at risk for cardiovascular disease; SMD, standardized mean difference.

Patients in the dialysis cohort (n = 15) who could not be matched were 31.2 ± 9.7 years old, 8 (53%) were men, 11 (73%) were treated with hemodialysis, 3 (20%) had diabetes mellitus, and none had a history of coronary artery disease.

### Cardiac CT Results

**Figure 1:**
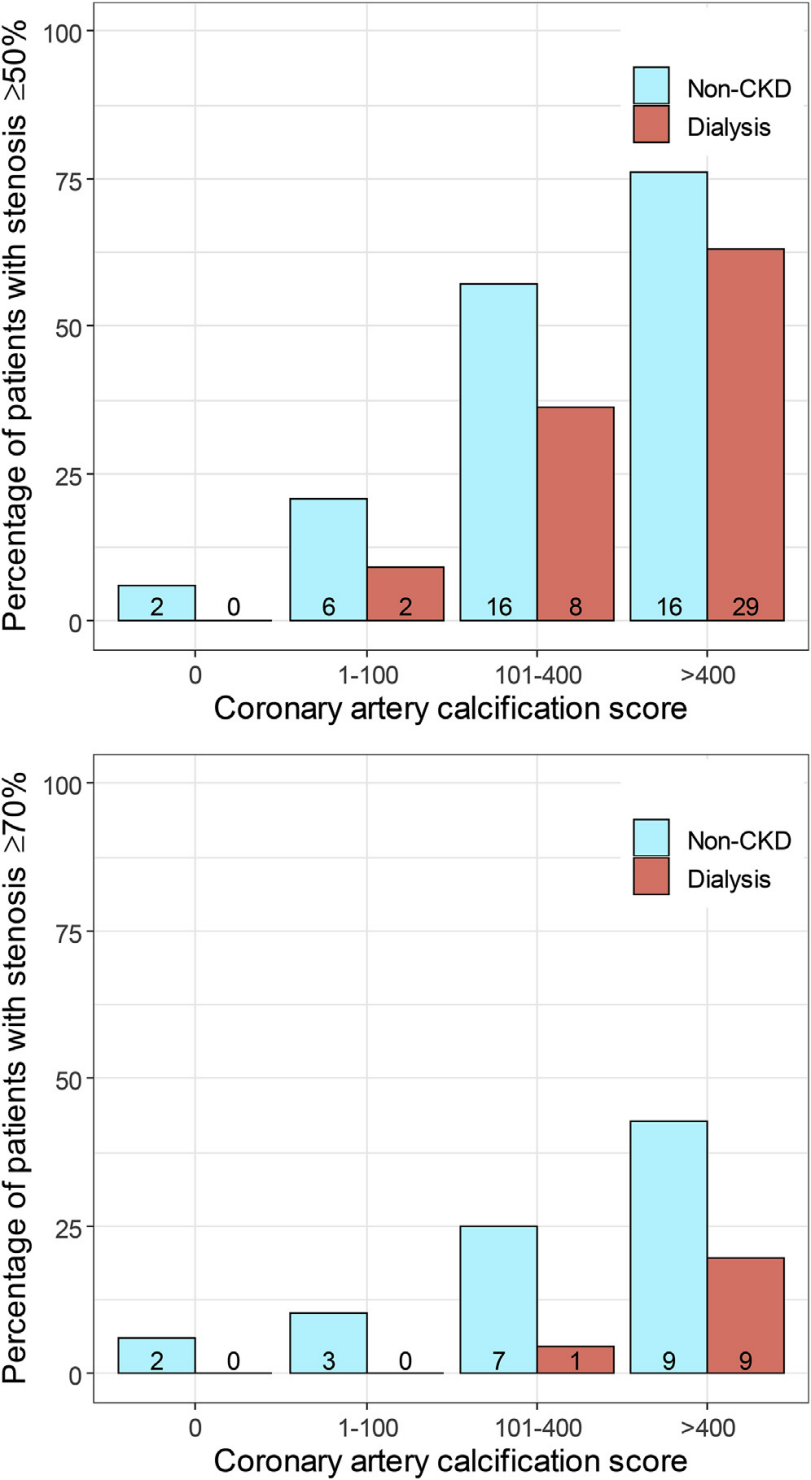
Percentage of patients with ≥50% and ≥70% coronary artery stenosis per category of coronary artery calcification (CAC) score in 112 dialysis patients and 112 propensity score–matched patients without chronic kidney disease (CKD). Number of patients provided in individual bars.

In the matched dialysis cohort, median CAC score was 210 (IQR, 19-859), 39 (35%) patients had stenosis ≥ 50%, and 10 (9%) patients had stenosis ≥ 70%. In the matched non-CKD cohort, median CAC score was 58 (IQR, 0-254), 40 (36%) patients had stenosis ≥ 50%, and 21 (19%) patients had stenosis ≥ 70% (Table 2; Fig 1).

**Figure 2:**
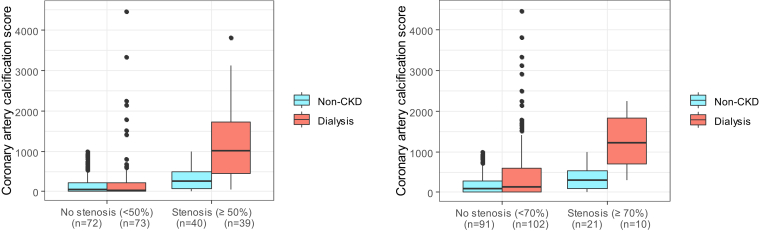
Comparison of coronary artery calcification score between matched dialysis patients and patients without chronic kidney disease (CKD).

Regarding the relationship between CAC and stenosis, patients without CAC were unlikely to have any stenosis ≥ 50% or ≥70%, whether they were in the dialysis cohort or the non-CKD cohort (Fig 1). However, higher CAC scores were observed in a considerable proportion of dialysis patients who did not have a stenosis ≥ 50% or ≥70% (Fig 2). In patients without CKD, higher CAC score was more prominently associated with stenosis. For example, of the dialysis patients and CAC score > 400, a total of 20% of patients had stenosis ≥ 70% compared with 43% in patients without CKD.

In the dialysis cohort, each 100-unit higher CAC score was associated with 1.12 and 1.02 times higher odds of the presence of stenosis ≥ 50% and ≥70%, respectively (95% CI, 1.06-1.20 and 95% CI, 0.98-1.06). In the non-CKD cohort, each 100-unit higher CAC score was associated with 1.68 and 1.37 times higher odds of the presence of stenosis ≥ 50% and ≥70%, respectively (95% CI, 1.37-2.14 and 95% CI, 1.14-1.65). The dialysis cohort had significantly lower odds ratios for stenosis than the non-CKD cohort, 0.67 (95% CI, 0.52-0.83) for stenosis ≥ 50% and 0.75 (95% CI, 0.62-0.90) for stenosis ≥ 70%.

**Figure 3:**
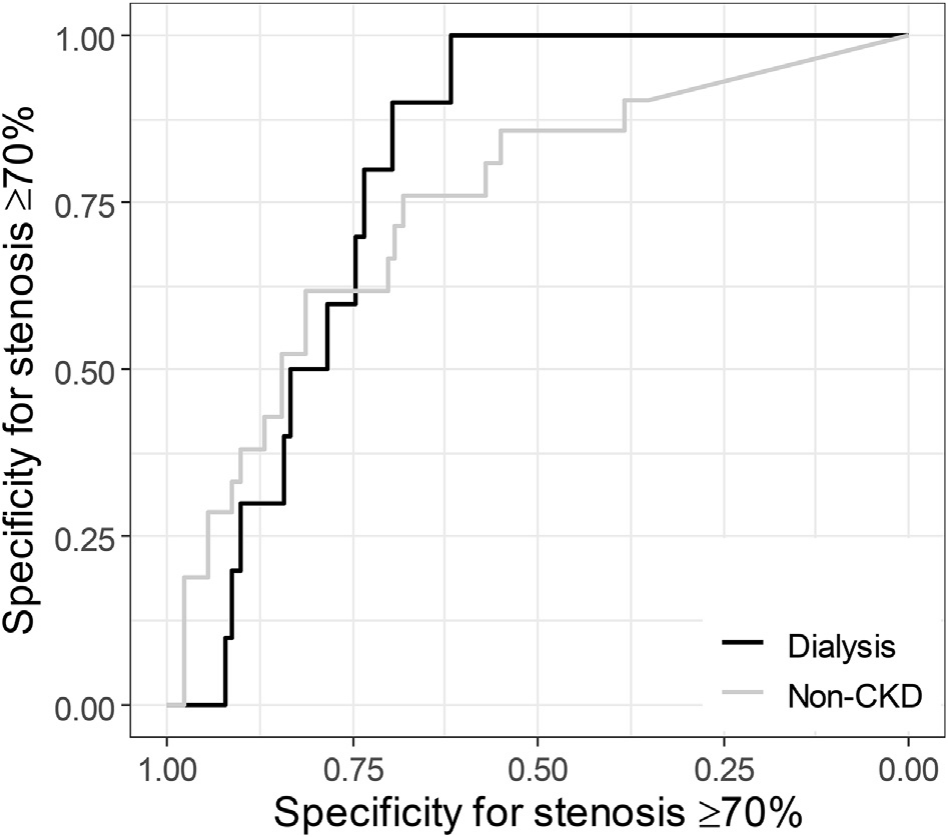
Receiver operating characteristics curve shows the discriminating ability of coronary artery calcification for coronary artery stenosis in dialysis patients and patients without chronic kidney disease (CKD).

To illustrate further, a cutoff value of CAC score > 400 in dialysis patients had sensitivity of 0.74 (95% CI, 0.5-0.87) and specificity of 0.77 (95% CI, 0.65-0.86) for detecting ≥50% stenosis, with a positive predictive value (PPV) of 0.63 (95% CI, 0.48-0.77) and negative predictive value (NPV) of 0.85 (95% CI, 0.74-0.92). In patients without CKD, sensitivity was 0.40 (95% CI, 0.25-0.57) and specificity was 0.93 (95% CI, 0.85-0.98), with a PPV of 0.76 (95% CI, 0.53-0.92) and NPV of 0.74 (95% CI, 0.63-0.82). A cutoff value of CAC score > 400 in dialysis patients for detecting ≥70% stenosis had sensitivity of 0.90 (95% CI, 0.56-1.00) and specificity of 0.64 (95% CI, 0.54-0.73), with PPV of 0.20 (95% CI, 0.09-0.34) and NPV of 0.98 (95% CI, 0.92-1.00), whereas in patients without CKD, sensitivity was 0.43 (95% CI, 0.22-0.66) and specificity was 0.87 (95% CI, 0.78-0.93), with a PPV of 0.43 (95% CI, 0.22-0.66) and NPV of 0.87 (95% CI, 0.78-0.93). Figure 3 shows the receiver operating characteristic curve for stenosis ≥ 70%.

Regarding individual lesions in coronary arteries, all except 1 patient provided an interpretable coronary CT angiography scan. We therefore excluded this patient (70-year-old woman with ESKD and CAC score of 1,408) from analyses. Patients in the dialysis and non-CKD cohorts had a similar (*P* = 0.49) number of lesions in total (Table 3). However, dialysis patients had significantly more calcified lesions (*P* = 0.01) and significantly fewer noncalcified lesions (*P* < 0.001) than patients in the non-CKD cohort. There was no significant difference in number of partially calcified lesions (*P* = 0.18) between patients in the dialysis and non-CKD cohorts.

**Table 3:** Coronary Computed Tomography Angiography Results in Propensity Score–Matched Cohorts of 112 Dialysis Patients and 112 Without Chronic Kidney Disease

	Dialysis (n = 112)	Non-CKD (n = 112)	*P*^a^
Interpretable scans^b^	111 (99%)	112 (100%)	0.99
With CABG	3 (3%)	8 (7%)	0.22
With coronary stent	7 (6%)	51 (46%)	<0.001
Without any lesions	18 (16%)	19 (17%)	0.99
Per patient^a^			
No. of coronary segments	14.0 ± 1.2	14.4 ± 1.0	0.03
Total no. of lesions	11.3 ± 8.6	10.5 ± 8.9	0.49
No. of noncalcified lesions	0.5 ± 1.0	2.4 ± 3.6	<0.001
No. of partially calcified lesions	5.8 ± 5.2	5.1 ± 5.5	0.18
No. of calcified lesions	5.0 ± 5.7	3.1 ± 4.0	0.01

*Note:* Results are presented as mean ±standard deviation or number (percent).

Abbreviations and Definitions: CABG, coronary artery bypass grafting; Non-CKD, non–chronic kidney disease at risk for cardiovascular disease.

a Significance tested with χ^2^ tests for proportions and Mann-Whitney *U* tests for number of lesions.

b In 1 patient, computed tomography angiography could not be interpreted reliably (see text).

### Secondary Analyses on the Full Cohort of Patients

These analyses were repeated in the entire dialysis (n = 127) and non-CKD (n = 447) cohorts, yielding similar results on the percentage of ≥50% and ≥70% stenosis and comparison of calcified and noncalcified lesions (Table S1). These also showed that dialysis patients had significantly lower odds ratios for stenosis than the non-CKD cohort when adjusted in multivariable analysis: 0.89 (95% CI, 0.79-1.00) for stenosis ≥ 50% and 0.87 (95% CI, 0.77-0.98) for stenosis ≥ 70% (Tables S2 and S3).

## Discussion

In an investigation of whether vascular calcification in dialysis patients reflects coronary artery stenosis to the same extent as in patients without CKD, we show that dialysis patients are less likely to have coronary artery stenosis as compared with patients without CKD with similar CAC scores. At equal CAC scores, the probability for significant stenosis in dialysis patients is about 1.5 times less than in patients without kidney disease. The lesions in coronary arteries were more often calcified in patients with ESKD compared with patients without CKD.

The background of this question lies in the fact that the generalized vessel wall calcification in patients with ESKD is assumed to be localized in the media. As such, the contribution of calcification noted on coronary CT to coronary artery stenosis is undetermined. This study thus helps quantify the complex relationship between multifocal calcification and obstructive coronary artery disease in patients with kidney failure.

How do these findings translate to the interpretation of CAC scores in dialysis patients? CAC scoring is generally used to rule out coronary artery stenosis because CAC has high sensitivity for coronary artery stenosis and patients without CAC are unlikely to have significant coronary artery stenosis. This is even more true in patients treated with dialysis: when a dialysis patient has a low CAC score, the probability for stenosis is lower than for a patient without CKD. However, our data show that even with CAC scores > 400, dialysis patients are unlikely to have coronary artery stenosis, whereas patients without CKD with CAC scores > 400 have a high risk for coronary artery stenosis. This means that CAC scores cannot readily be used to infer obstructive coronary artery disease risk in patients receiving dialysis.

These findings complement the current literature by providing insight into the diagnostic capacity of CT angiography in patients treated with dialysis. In patients without CKD, the diagnostic performance of coronary CT angiography for significant stenosis is well established, although it is accepted that the diagnostic capacity of CT angiography diminishes with increased calcification burden.^28^ However, many CT angiography studies in patients with CKD are limited to patients with relatively preserved kidney function (GFR > 30 mL/min).^29^ Although in patients with GFRs of 30 to 60 mL/min, increased CAC scores are noted,^30^ results cannot be extrapolated to the dialysis population because only in ESKD do hyperphosphatemia and severe calcification occur. Some studies include both patients with CKD and patients receiving dialysis, making it difficult to draw a firm conclusion for either group.^31^

Only a few studies specifically investigated patients with ESKD: a small study in 18 dialysis patients found no relationship between calcification and stenosis^21^ but likely was underpowered. Moreover, a somewhat larger study comparing 48 patients with ESKD with 68 patients without ESKD established a good correlation of CAC score and plaque burden.^20^ However, patients were selected on the basis of angina symptoms, leading to inclusion bias due to overrepresentation of intimal stenosis in this cohort. Importantly, this study did not correct for the traditional risk factors of cardiovascular disease. Interestingly, the authors found more dense calcifications in ESKD,^20^ possibly explaining the diminished risk for plaque rupture found in patients with CKD as kidney function decreases.^32^ A remarkable finding in our study was that despite very high CAC scores, the percentage of ≥50% and ≥70% stenosis in dialysis patients was relatively low (respectively, 63% and 20% of patients with CAC scores > 400). This supports the assertion that a large part of the calcium is located in the media in patients treated with dialysis. Nevertheless, this assertion is yet to be supported by histologic evidence.

Our study has several strengths. It is the first to quantify the discordance between calcification and coronary artery stenosis in dialysis patients compared with patients without kidney disease. Furthermore, we used 2 different techniques to control for potentially confounding variables relative to the sample size. First, we constructed 2 comparable patient cohorts based on their propensity score to develop ESKD. In our opinion, this method is a way to compare groups that are very difficult to compare in traditional ways because dialysis patients are in many aspects very different from patients without CKD. We could have performed “propensity score adjustment,” including the propensity score as a covariate in a multivariable regression model instead of propensity score matching.^33^ However, our dialysis group contained some young patients who were not in any way comparable to patients without CKD. With this technique, we compared the patients who can be compared.

Second, we performed an analysis of the entire cohort and tested for interaction between the presence of dialysis and coronary calcification on their effect on stenosis. Because this interaction was present, we reported the results for the dialysis and non-CKD groups separately. This additional analysis confirmed our original findings.

Other strengths are that our patient cohort is representative because most patients with ESKD had been receiving dialysis for more than 2 years. Thus, the included patients had a relatively large exposure to the determinant of dialysis. Also, this is the largest cohort of its kind and CAC score determination and coronary CT angiography were performed in a single center.

Our study also has some limitations. We could not compare cardiovascular end points in this study. Future studies should compare the associations of CAC scores and cardiac events between patients with and without ESKD, especially because it seems possible to attenuate the progression of coronary calcium.^34^ Furthermore, our non-CKD cohort was selected from a cohort of patients with risk factors for or established cardiovascular disease. This might have influenced the results. However, comparison with a healthy patient group would have been unfeasible because the prevalence of vascular calcification is very low in unselected healthy persons. Also, the inclusion criterion was GFR > 45 mL/min for patients without CKD, so in theory, patients could have had CKD stages 1-3a. However, because mean GFR was 91 mL/min, we are confident that most patients in the non-CKD group had near-normal kidney function.

A methodological limitation could be that we did not consider the presence of a stent as proof of a (former) coronary stenosis. Stents were more prevalent in the non-CKD than in the dialysis population. We did not include the stented segments in the analysis of stenosis. If we had considered a stent as proof of stenosis, this would have enforced our conclusions because we would have found even more stenoses in the non-CKD group.

Finally, this study investigated asymptomatic dialysis patients and asymptomatic patients without CKD. One could argue that this would not be relevant for clinical practice; however, this adds meaning to the (incidental) finding of vascular calcification in whichever patient being associated with higher risk for stenosis in a patient without CKD compared with a dialysis patient.

In conclusion, in patients receiving dialysis, each 100-unit higher CAC score is about 1.5 times less often associated with coronary artery stenosis than in patients without CKD with comparable risk factors for cardiovascular disease. When CAC scores greater than 400 in patients without CKD reflect a high probability of coronary artery stenosis, this is different in dialysis patients, in whom high CAC scores can easily be found without significant coronary artery stenosis.

## References

[1] Erbel R., Mohlenkamp S., Moebus S. (2010). Coronary risk stratification, discrimination, and reclassification improvement based on quantification of subclinical coronary atherosclerosis: the Heinz Nixdorf Recall study. J Am Coll Cardiol.

[2] Blaha M.J., Budoff M.J., DeFilippis A.P. (2011). Associations between C-reactive protein, coronary artery calcium, and cardiovascular events: implications for the JUPITER population from MESA, a population-based cohort study. Lancet.

[3] Saran R., Li Y., Robinson B. (2016). US Renal Data System 2015 Annual Data Report: epidemiology of kidney disease in the United States. Am J Kidney Dis.

[4] Goodman W.G., Goldin J., Kuizon B.D. (2000). Coronary-artery calcification in young adults with end-stage renal disease who are undergoing dialysis. N Engl J Med.

[5] Raggi P., Boulay A., Chasan-Taber S. (2002). Cardiac calcification in adult hemodialysis patients. A link between end-stage renal disease and cardiovascular disease?. J Am Coll Cardiol.

[6] Sigrist M.K., Taal M.W., Bungay P., McIntyre C.W. (2007). Progressive vascular calcification over 2 years is associated with arterial stiffening and increased mortality in patients with stages 4 and 5 chronic kidney disease. Clin J Am Soc Nephrol.

[7] Budoff M.J., Young R., Lopez V.A. (2013). Progression of coronary calcium and incident coronary heart disease events: MESA (Multi-Ethnic Study of Atherosclerosis). J Am Coll Cardiol.

[8] Bellasi A., Ferramosca E., Ratti C., Block G., Raggi P. (2016). The density of calcified plaques and the volume of calcium predict mortality in hemodialysis patients. Atherosclerosis.

[9] Sakaguchi Y., Hamano T., Nakano C. (2016). Association between density of coronary artery calcification and serum magnesium levels among patients with chronic kidney disease. PLoS One.

[10] Molenaar F.M., van Reekum F.E., Rookmaaker M.B., Abrahams A.C., van Jaarsveld B.C. (2014). Extraosseous calcification in end-stage renal disease: from visceral organs to vasculature. Semin Dial.

[11] Vervloet M., Cozzolino M. (2017). Vascular calcification in chronic kidney disease: different bricks in the wall?. Kidney Int.

[12] Blacher J., Guerin A.P., Pannier B., Marchais S.J., London G.M. (2001). Arterial calcifications, arterial stiffness, and cardiovascular risk in end-stage renal disease. Hypertension.

[13] Oh J., Wunsch R., Turzer M. (2002). Advanced coronary and carotid arteriopathy in young adults with childhood-onset chronic renal failure. Circulation.

[14] Wang A.Y., Wang M., Woo J. (2003). Cardiac valve calcification as an important predictor for all-cause mortality and cardiovascular mortality in long-term peritoneal dialysis patients: a prospective study. J Am Soc Nephrol.

[15] Nguyen P.T., Henrard S., Coche E., Goffin E., Devuyst O., Jadoul M. (2010). Coronary artery calcification: a strong predictor of cardiovascular events in renal transplant recipients. Nephrol Dial Transplant.

[16] Rumberger J.A., Sheedy P.F., Breen J.F., Schwartz R.S. (1995). Coronary calcium, as determined by electron beam computed tomography, and coronary disease on arteriogram. Effect of patient’s sex on diagnosis. Circulation.

[17] Haberl R., Becker A., Leber A. (2001). Correlation of coronary calcification and angiographically documented stenoses in patients with suspected coronary artery disease: results of 1,764 patients. J Am Coll Cardiol.

[18] Moe S.M., O’Neill K.D., Duan D. (2002). Medial artery calcification in ESRD patients is associated with deposition of bone matrix proteins. Kidney Int.

[19] Nallamothu B.K., Saint S., Bielak L.F. (2001). Electron-beam computed tomography in the diagnosis of coronary artery disease: a meta-analysis. Arch Intern Med.

[20] Jug B., Kadakia J., Gupta M. (2013). Coronary calcifications and plaque characteristics in patients with end-stage renal disease: a computed tomographic study. Coron Artery Dis.

[21] Sharples E.J., Pereira D., Summers S. (2004). Coronary artery calcification measured with electron-beam computerized tomography correlates poorly with coronary artery angiography in dialysis patients. Am J Kidney Dis.

[22] van ‘t Klooster C.C., Nathoe H.M., Hjortnaes J. (2020). Multifocal cardiovascular calcification in patients with established cardiovascular disease; prevalence, risk factors, and relation with recurrent cardiovascular disease. Int J Cardiol Heart Vasc.

[23] Abbara S., Blanke P., Maroules C.D. (2016). SCCT guidelines for the performance and acquisition of coronary computed tomographic angiography: a report of the society of Cardiovascular Computed Tomography Guidelines Committee: Endorsed by the North American Society for Cardiovascular Imaging (NASCI). J Cardiovasc Comput Tomogr.

[24] Leipsic J., Abbara S., Achenbach S. (2014). SCCT guidelines for the interpretation and reporting of coronary CT angiography: a report of the Society of Cardiovascular Computed Tomography Guidelines Committee. J Cardiovasc Comput Tomogr.

[25] Simons P.C., Algra A., van de Laak M.F., Grobbee D.E., van der Graaf Y. (1999). Second manifestations of ARTerial disease (SMART) study: rationale and design. Eur J Epidemiol.

[26] Dorresteijn J.A., Visseren F.L., Wassink A.M. (2013). Development and validation of a prediction rule for recurrent vascular events based on a cohort study of patients with arterial disease: the SMART risk score. Heart.

[27] Ho D.E., Imai K., King G., Stuart E.A. (2011). MatchIt: nonparametric preprocessing for parametric causal inference. J Stat Softw.

[28] den Dekker M.A., de Smet K., de Bock G.H., Tio R.A., Oudkerk M., Vliegenthart R. (2012). Diagnostic performance of coronary CT angiography for stenosis detection according to calcium score: systematic review and meta-analysis. Eur Radiol.

[29] Joosen I.A., Schiphof F., Versteylen M.O. (2012). Relation between mild to moderate chronic kidney disease and coronary artery disease determined with coronary CT angiography. PLoS One.

[30] Chen J., Budoff M.J., Reilly M.P. (2017). Coronary artery calcification and risk of cardiovascular disease and death among patients with chronic kidney disease. JAMA Cardiol.

[31] Haydar A.A., Hujairi N.M., Covic A.A., Pereira D., Rubens M., Goldsmith D.J. (2004). Coronary artery calcification is related to coronary atherosclerosis in chronic renal disease patients: a study comparing EBCT-generated coronary artery calcium scores and coronary angiography. Nephrol Dial Transplant.

[32] Chin C.Y., Mintz G.S., Saito S. (2017). Relation between renal function and coronary plaque morphology (from the Assessment of Dual Antiplatelet Therapy With Drug-Eluting Stents Virtual Histology-Intravascular Ultrasound Substudy). Am J Cardiol.

[33] Fu E.L., Groenwold R.H.H., Zoccali C., Jager K.J., van Diepen M., Dekker F.W. (2019). Merits and caveats of propensity scores to adjust for confounding. Nephrol Dial Transplant.

[34] Raggi P., Bellasi A., Bushinsky D. (2020). Slowing progression of cardiovascular calcification with SNF472 in patients on hemodialysis: results of a randomized phase 2b study. Circulation.

