# Supplementary material for: Coronary Artery Calcification as a Marker for Coronary Artery Stenosis: Comparing Kidney Failure to the General Population
Source: Kidney Med. 2021 Apr 20;3(3):386–394.e1. doi: 10.1016/j.xkme.2021.01.010 (PMC8178454; doi:10.1016/j.xkme.2021.01.010)
Supplement: Supplementary File (PDF) — Table S1-S3. [file mmc1.pdf]

Table S1. Coronary computed tomography angiography results and calcification scores in 127 dialysis patients and 447 patients without chronic kidney disease.

	**Dialysis (n=127)**	**Non-CKD (n=447)**	***P-value*****
**Interpretable scans* (%)**	126 (99%)	437 (98%)	0.99
**With CABG (%)**	3 (2%)	47 (11%)	0.01
**With coronary stent (%)**	7 (6%)	208 (47%)	<0.001
**Without any lesions (%)**	29 (23%)	50 (11%)	<0.01
**CAC score**	149 (0 – 748)	105 (3 – 324)	0.04
**With coronary stenosis (%)**			
• **≥50%**	40 (31%)	157 (35%)	0.45
• **≥70%**	10 (8%)	79 (18%)	0.01
Per patient			
**Number of coronary segments**	14.0 ±1.4	14.0 ±1.3	0.78
**Total number of lesions**	11.7 ±8.7	10.3 ±8.6	0.10
• **Number of non-calcified lesions**	0.4 ±1.0	2.5 ±3.7	<0.001
• **Number of partially calcified lesions**	5.3 ±5.2	5.2 ±5.0	0.97
• **Number of calcified lesions**	4.6 ±5.6	4.0 ±4.2	0.83

Results are presented as mean ±standard deviation or number (percentage). Abbreviations: Non-CKD: non-chronic kidney disease at risk for cardiovascular disease; CABG: coronary artery bypass grafting.

* In one patient CT angiography could not be interpreted reliably (see text).

** Significance tested with Chi-squared tests for proportions and Mann-Whitney-U tests for number of lesions.

Table S2. Relationship of coronary artery calcification score with ≥50% coronary artery stenosis on coronary computed tomography angiography, and interaction of dialysis with this relationship in 127 dialysis patients and 447 patients without chronic kidney disease.

	**Odds ratio (95% CI)**	***P-value***
**Odds ratio of ≥50% stenosis per 100 unit higher CAC score (unadjusted)**		
• **ESKD**	1.14 (1.08–1.21)	<0.001
• **Non-CKD**	1.32 (1.22–1.44)	<0.001
• **Interaction term for ESKD compared with non-CKD**	0.86 (0.78–0.95)	<0.01
**Odds ratio of ≥50% stenosis per 100 unit higher CAC score (adjusted*)**		
• **ESKD**	1.15 (1.07–1.25)	0.001
• **Non-CKD**	1.29 (1.19–1.42)	<0.001
• **Interaction term for ESKD**	0.89 (0.79–1.00)	0.05

*Adjusted for age, sex, body mass index, current smoking, presence of diabetes mellitus, C-reactive protein and total cholesterol.

Table S3. Relationship of coronary artery calcification score with ≥70% coronary artery stenosis on coronary computed tomography angiography, and interaction of end-stage kidney disease with this relationship in 127 patients with end-stage kidney disease and 447 without chronic kidney disease.

	**Odds ratio (95% CI)**	***P-value***
**Odds ratio of ≥70% stenosis per 100 unit higher CAC score (unadjusted)**		
• **ESKD**	1.03 (0.98–1.06)	0.15
• **Non-CKD**	1.24 (1.13–1.35)	<0.001
• ***Interaction term for ESKD compared with non-CKD***	*0.83 (0.76–0.91)*	*<0.001*
**Odds ratio of ≥70% stenosis per 100 unit higher CAC score (adjusted*)**		
• **ESKD**	1.06 (0.98–1.15)	0.13
• **Non-CKD**	1.22 (1.11–1.34)	<0.001
• ***Interaction term for ESKD***	*0.87 (0.77–0.98)*	*0.02*

*Adjusted for age, sex, body mass index, current smoking, presence of diabetes mellitus, C-reactive protein and total cholesterol.

Abbreviations: CAC coronary artery calcification; ESKD end-stage kidney disease; Non-CKD non-chronic kidney disease.
